# New Approach to Shape Memory Polymer Composite Production Using Alkaline Lignin-Reinforced Epoxy-Based Shape Memory Polymers

**DOI:** 10.1021/acsomega.2c07812

**Published:** 2023-04-20

**Authors:** Merve Uyan, Melih Soner Celiktas

**Affiliations:** † Solar Energy Institute, Ege University, Bornova, Izmir 35100, Turkey; ‡ Department of Mechanical Engineering, University of Alberta, Edmonton, Alberta T6G 2R3, Canada

## Abstract

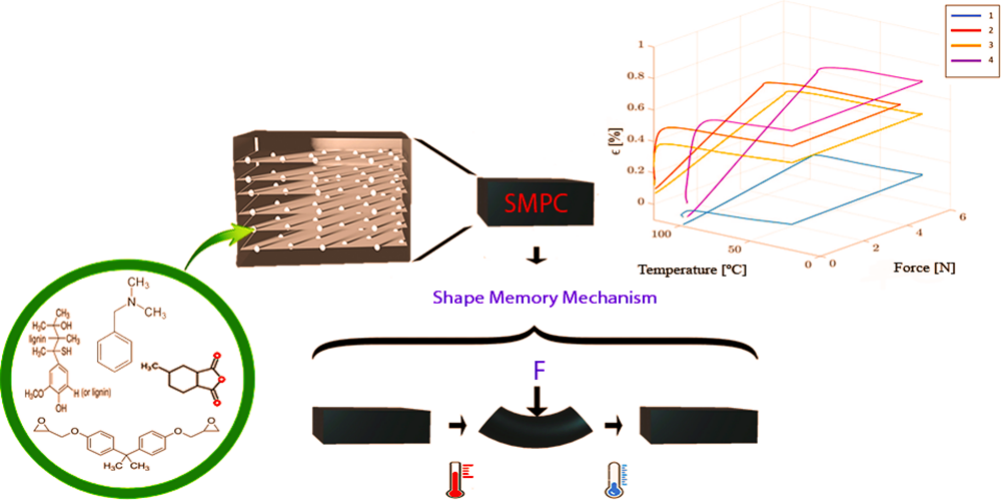

In the past few decades, there has been continued interest in shape memory polymers (SMPs), and tremendous efforts have been made to develop multifunctional composites of these SMPs to enhance the existing properties of SMPs. Although fossil-based sources are widely used in the production of shape memory polymer composites (SMPCs), the depletion of fossil-based resources and associated environmental problems increase interest toward renewable biobased products synthesized from natural resources. This study aims to produce alkaline lignin-reinforced SMPCs by using alkaline lignin in the SMP matrix. Thermo-mechanical, morphological, and shape memory tests are performed in order to reveal the effect of alkaline lignin usage in the SMP matrix on SMPC production. Differential scanning calorimetry analysis results show that adding alkaline lignin into the SMP matrix with 1 and 3% ratios led to an increase in *T*_g_ values, while raising the alkaline lignin ratio to 5% decreased the *T*_g_ value. According to the DMA results, increasing the alkaline lignin ratios caused an increase in the storage modulus of SMPCs, and the best storage modulus value was obtained at the 5% alkaline lignin ratio. The results of the three-point bending test also confirmed the results obtained from the DMA analysis, showing that an increasing alkaline lignin ratio caused an increase in the bending modulus. Scanning electron microscopy analysis showed a rough structure in 1 and 3% alkaline lignin supplementation, while a smoother structure was observed in 5% alkaline lignin supplementation. The smoother structure of the sample containing 5% alkaline lignin indicates that alkaline lignin supplementation exhibits a smoother surface by showing a plasticizing effect. As a result, it was observed that increasing the lignin ratio increased the polymer/alkaline lignin interaction, resulting in a harder structure and an increase in the flexural modulus value.

## 1 Introduction

Shape memory polymers (SMPs) are known as smart materials. They have capabilities to change their shape under different stimuli such as heat, light, microwave, electricity, and water.^1−3^ The properties of SMPs such as easy deformability, adjustable *T*_g_ values, lightweight, and high shape recoverability allow an extensive scope of application from aerospace to biomedical.^4−6^ SMPs can be classified as thermoset and thermoplastic SMPs.^7,8^ The main difference between thermoset and thermoplastic SMPs is their crosslinking structure.^9^

Thermoset SMPs have a covalent crosslinking structure, while thermoplastic SMPs have a physical crosslinking structure. Due to their covalent crosslinking structure, thermoset SMPs are stronger than thermoplastic SMPs.^10^ Their transition temperature depends on the crosslinking structure. In thermoset SMPs, transition temperature is known as glass transition temperature (*T*_g_), whereas in thermoplastic SMPs, transition temperature is known as melting temperature (*T*_m_).^7^ The transition temperatures have a significant effect on activating the shape memory mechanisms of thermally activated SMPs. The activation mechanism of SMPs must be adjusted with considering their transition temperature. The first step to activate the thermoset SMPs is heating the SMPs above their *T*_g_ value. Then, external force is applied to SMPs in order to create deformation. The third step is reducing the temperature under their *T*_g_ values under constant load. The final step is removing the external force and heating the SMPs over their *T*_g_ value in order to complete the shape memory mechanism. The difference in the shape memory mechanism for thermoplastic SMPs is their switching temperature.

The past decades have witnessed studies to improve the SMP properties to enhance their usage in various application areas. Additive materials are used for improving their properties. These additive materials comprise short and long fibers, particles, etc. There is increasing interest in improving SMP properties, and SMPCs are gaining attention.^3,11,12^ SMPCs are produced with the addition of different additive materials into the SMP structure. They have desired properties such as excellent mechanical strength and thermal properties for advanced applications.^6,13,14^ Additive materials have great influence on the final properties of SMPCs. Short and long fiber-reinforced SMPCs have large application areas.^15−18^

Generally, fossil-based materials are used as matrix and reinforcement materials in SMPC production.^19,20^ Increasing environmental concern has led to research for using renewable sources in SMPC production.^21,22^ Biobased polymers can be incorporated into the fossil-based shape memory polymer structure used directly as a matrix material for SMPCs. Although biobased SMPs produced from renewable sources are promising for usage as matrix materials, they have some limitations due to their hydrophilic structural properties.^23,24^ Their hydrophilic structure results in low interface adhesion with the hydrophobic matrix in which they are incorporated.^25^ The low interface adhesion has a significant impact on the mechanical and thermal properties of SMPCs.^26^

Investigation of the properties of biobased SMP-based composites for different applications has become a recent topic of great interest by researchers.^27−30^ Therefore, many studies have been carried out in order to obtain a good interface adhesion by improving the disadvantages of biobased polymers.^16,20,31−33^

Lignin is the second most abundant biopolymer on Earth after cellulose.^34,35^ It is produced as a byproduct in the pulp industries by various processes such as sulfites, kraft, and soda.^36,37^ Lignin has an amorphous structure and polar functional groups such as phenolic −OH, aliphatic hydroxyl, and carbonyl groups. These chemical bondings of lignin vary depending on the plant species from which they are obtained and the separation process used.^38,39^

Lignin used in commercial applications can be physically extracted from biomass by chemical or biochemical methods. They can be produced by sulfur-free processes such as alkaline pulping (soda lignin) and solvent pulping (organosolv lignin) in addition to sulfur-based processes such as kraft and sulfide pulping. Lignin types produced by different processes can be preferred in different applications because they have different molecular masses and phenolic and aliphatic hydroxyl groups.^40,41^

This chemical structure of the lignin used affects its solubility in the polymer it is included in and the bonds formed between the polymer/lignin. The incompatibility of the used lignin and the polymer causes agglomerations in the polymer structure, causing the polymer to have a brittle structure.^42,43^

Studies focusing on the use of lignin as an additive, in order to obtain low-cost and environmentally friendly polymer blends from lignin obtained from different processes, are ongoing.^40,44^

The complex chemical structure has made lignin a remarkable material for use in different polymeric systems. Many studies have been carried out in the literature on lignin–polymer mixtures, and the effects of lignin supplementation on the properties of the polymer have been revealed.^45,46^ These previous studies have shown that lignin can be used as a coupling agent in polymer composites. In recent years, studies on the use of lignin–polymer mixtures in the production of composite materials have increased, and the effects of lignin supplementation on the mechanical properties of composite materials have been revealed.^28,45^

Studies have shown that lignin/polymer mixtures have low mechanical properties, especially at high lignin loading ratios. However, it is stated that 10% or less lignin reinforcement causes an improvement in the mechanical properties of the obtained polymer mixture. Different amine curing agents and anhydride hardeners can be used to ensure that polymer mixtures prepared with lignin have good mechanical properties. These reinforcing materials increase the bonding between lignin and polymer, resulting in an improvement in the properties of the obtained polymer mixture. Thus, the usage potential of polymer blends obtained by incorporating lignin into polymer blends in certain proportions for the production of composite materials is increased. In addition, by reducing the amount of fossil-based polymers used in the matrix phase in composite material production, the use of biobased products is increased.^28^

It has been reported that lignin increases the thermal stability of the polymer and has a plasticizing effect on the polymer.^47^ Different lignin types are utilized in preparation of polymer blends for composite production. Zhu et al. found out that alkaline lignin from kraft and soda pulp gave the best results as a coupling agent for polymer-based composites compared to all other lignin types.^34^

Li et al. stated that it is difficult for epoxy resin mixtures obtained with a high lignin content to have mechanical properties comparable to synthetic epoxy resins, even though lignin has uses such as aromatic chemicals and an epoxy resin reinforcement material. In their study, the effects of lignosulfonate (LS) and ethylene glycol (EG) together on the properties of epoxy resin were investigated. They emphasized that lignin supplementation at low rates improved the properties of the epoxy resin.^48^ Zhang et al. prepared an SMP mixture with carboxylated lignosulfonate by combining diglycidyl ether of bisphenol-A (DGEBA) with poly(ethylene glycol) diglycidyl ether (PEGDGE) and polyetheramine (PEA). They indicated that carboxylated lignosulfonate incorporation results in a significant increase in the mechanical properties of the neat SMP mixture.^49^ Xu et al. synthesized polyester thermosets with lignin, PEG400, and citric acid. The obtained results demonstrated that lignin-based thermosets have a good shape fixity ratio of 95% and shape recovery ratio of 99%.^50^

In the current study, SMPCs were produced by the vacuum-assisted hand lay-up method using SMP containing alkaline lignin at different ratios (% 0, 1, 3, and 5) as the matrix phase and glass fiber as the reinforcement element. In comparison to other studies in the literature, the current study takes a comprehensive approach to reveal the effects of the interface formed by the SMP mixture and alkaline lignin on the thermo-mechanical, morphological, and shape memory properties of the produced glass fiber-reinforced SMPCs. Hence, this study differs from other studies in the literature in terms of presenting both the reinforcing and toughening effects of lignin, which has a rigid and highly branched structure, on glass fiber-reinforced SMPCs produced. Consequentially, the current study paved the way for the use of biobased materials in the matrix phase of SMPC production by demonstrating the potential of lignin, a byproduct of thermochemical processes, in SMPC production.

## 2 Materials and Method

**Figure 1:**
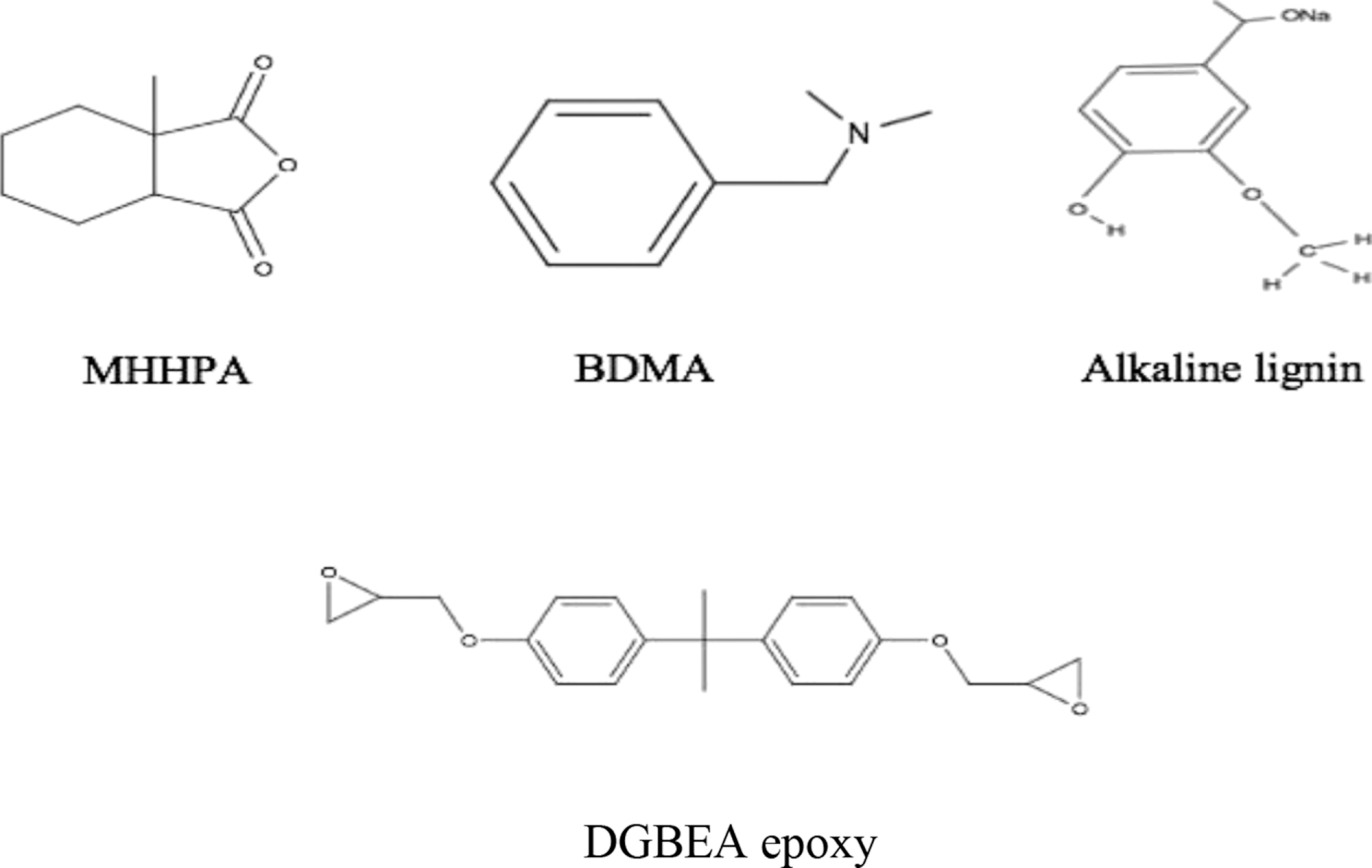
Chemical structures of DGBEA epoxy (E51), alkaline lignin, MHHPA, and BDMA.

Bisphenol, a diglycidyl ether (DGBEA, E51), methyl hexahydrophthalic anhydride (MHHPA), and benzyldimethylamine (BDMA) were purchased from ISOLAB. Alkaline lignin was purchased TOKYO CHEMICAL INDUSTRY. Glass fibers are purchased from FIBERMAK. The chemical structures of the purchased materials are shown in Figure 1.

### 2.1 Production of Shape Memory Epoxy Resin

In order to prepare shape memory epoxy resin, E 51 is heated to 60 °C in an oil bath. Then MHHPA is added into E51 with the determined ratio as 1:1 (w/w). After that, BDMA is added into solution with %1 of MHHPA. The solution is prepared by mixing with a mechanical stirrer. Finally, %0 alkaline lignin shape memory epoxy resin is prepared.

In the second step, 1, 3, and 5% alkaline lignin included shape memory epoxy resin mixtures are prepared. Shape memory epoxy resin mixtures with 1, 3, and 5% alkaline lignin ratios are calculated based on the mass of shape memory epoxy resin.

**Figure 2:**
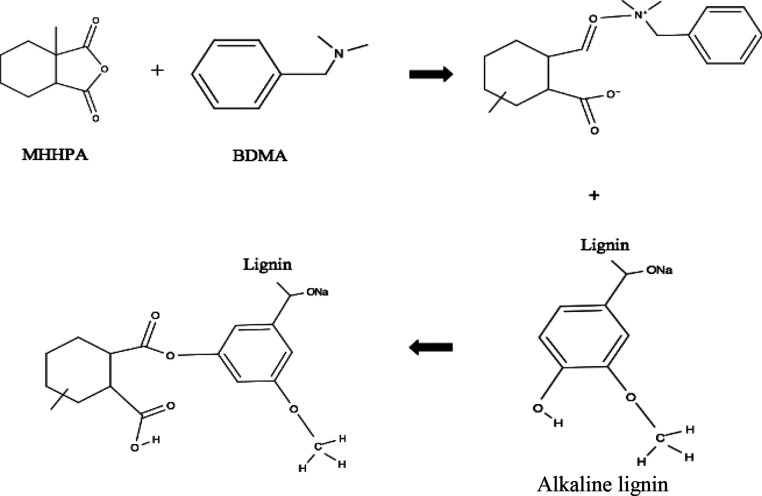
Schematic representation of the reaction mechanism of MHHPA with BDMA and MHHPA/BDMA with alkaline lignin.

**Figure 3:**
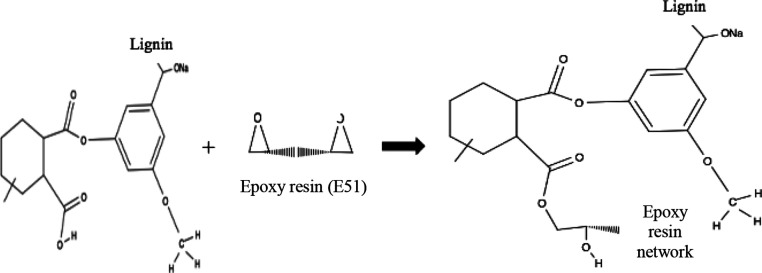
Schematic representation of the reaction procedure of MHHPA/BDMA/alkaline lignin with the E51.

The intermolecular bonds expected to be formed are the bonds between the anhydride used and the shape memory epoxy resin, and the bonds between the alkaline lignin and the epoxy resin. The reaction mechanisms between the MHHPA, BDMA, alkaline lignin, and E51 are shown in Figures 2 and 3.

### 2.2 Production of SMPCs

The prepared shape memory epoxy resin mixtures are used as matrix materials in the production of glass fiber-reinforced SMPCs. In the production of shape memory composite materials, the fiber ratio was determined as 30%.

In the production of SMPCs, six layer glass fibers were prepared at a dimension of 30 × 30 cm. First, a hand lay-up method is used to produce SMPCs on the vacuum infusion device. Then the vacuum nylon is covered on top of the layers and sealed with a vacuum band. Vacuum is applied for 15 min to remove air bubbles. At the end of the vacuum step, the prepared samples were cured at 80 °C on the vacuum infusion device.

## 3 Testing Methods

### 3.1 Differential Scanning Calorimetry (DSC) Analysis

Thermal properties of the SMPCs are determined utilizing a DSC device with nitrogen as purge gas. The SMPC samples are prepared for analysis according to ASTM D3418. The SMPC sample was cooled to 0 °C at a cooling rate of 10 C/min and then heated from 0 to 250 °C.

### 3.2 DMA Analysis

Thermo-mechanical properties of SMPCs are determined using a dynamic mechanical analyzer (DMA) Q800 device. The SMPC samples are prepared for analysis according to ASTM D7028. The temperature is scanned from 25 to 160 °C. DMA tests were carried out at a frequency of 1 Hz and a strain value of 0.023%. The storage modulus, tan delta, loss modulus are determined with DMA analysis.

### 3.3 Scanning Electron Microscopy/Energy-Dispersive Spectroscopy (SEM/EDS) Analysis

The morphological properties are determined using SEM/EDS analysis. The SMPC samples were coated with Au before analysis. The surface morphology and the distribution of fibers in the matrix phase were examined with SEM analysis. The calibrative elemental analysis was conducted with EDS analysis.

### 3.4 FTIR Analysis

FTIR analyses were carried out on an iS50 FTIR device to observe the chemical bond formations between the shape memory polymer mixture and alkaline lignin used during the production of SMPCs and to determine the changes in these bonds for different ratios of alkaline lignin.

### 3.5 Three-Point Bending Test

The flexural properties of SMPCs were determined by the three-point bending test. The SMPC samples are prepared for analysis according to ASTM D 7264. The flexural modulus and maximum strength values are obtained from the three-point bending test.

### 3.6 Shape Memory Tests

Shape fixity and shape recovery ratio tests are performed in the controlled force mode of the DMA (Q800) device. The respective DMA program begins with a temperature ramp of 5 °C/min up to (*T*_g_ + 20) °C, continued by a constant temperature of (*T*_g_ + 20) °C for 10 min, and force ramp of 0.5 N/min to 6 N continued by a temperature ramp of 5 °C/min down to 20 °C followed by a constant temperature with 6 N constant force for 20 min. The deformed strain is also labeled as ε_max_. Applied force is then released with a ramp of 6 N/min to 0.001 N and continued at constant temperature for 10 min to achieve a temporary fixed shape, and the fixed strain is labeled as ε_fix_. Finally, the sample was subjected to a temperature ramp of 5 °C/min up to (*T*_g_ + 20) °C and isothermal for 20 min, and the shape recovery was achieved (ε_recovery_). The shape fixity and recovery ratios can be calculated by eqs 1 and 2.
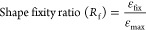
1
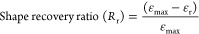
2

## 4 Results and Discussion

### 4.1 DSC Analysis

**Figure 4:**
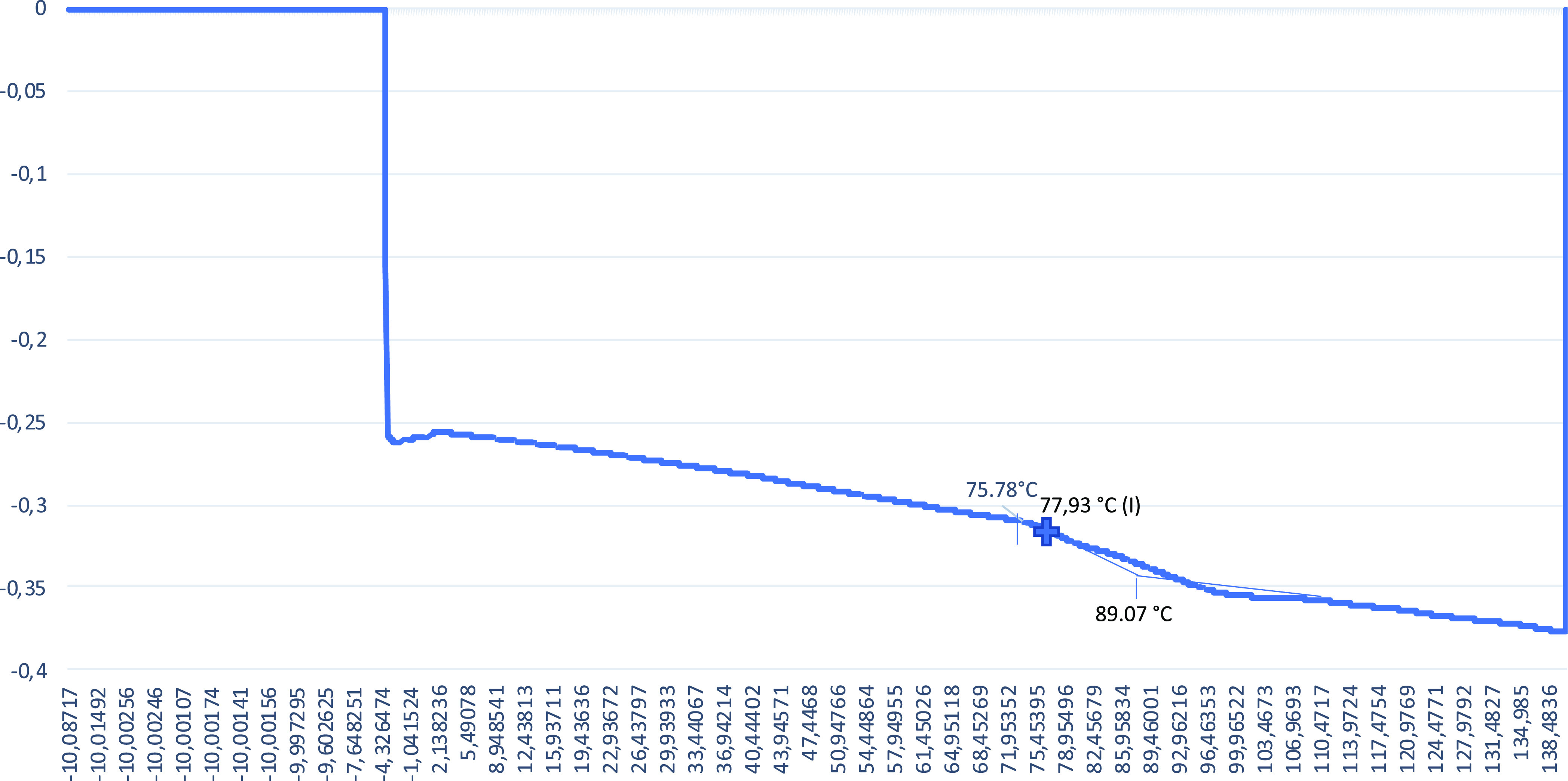
DSC graph for the 0% alkaline lignin included sample.

The obtained *T*_g_ values are given in Figures 4^5^,6,7 from DSC analysis. According to results, the incorporation of the alkaline lignin into the SMP structure has a significant effect on the *T*_g_ values.

**Figure 5:**
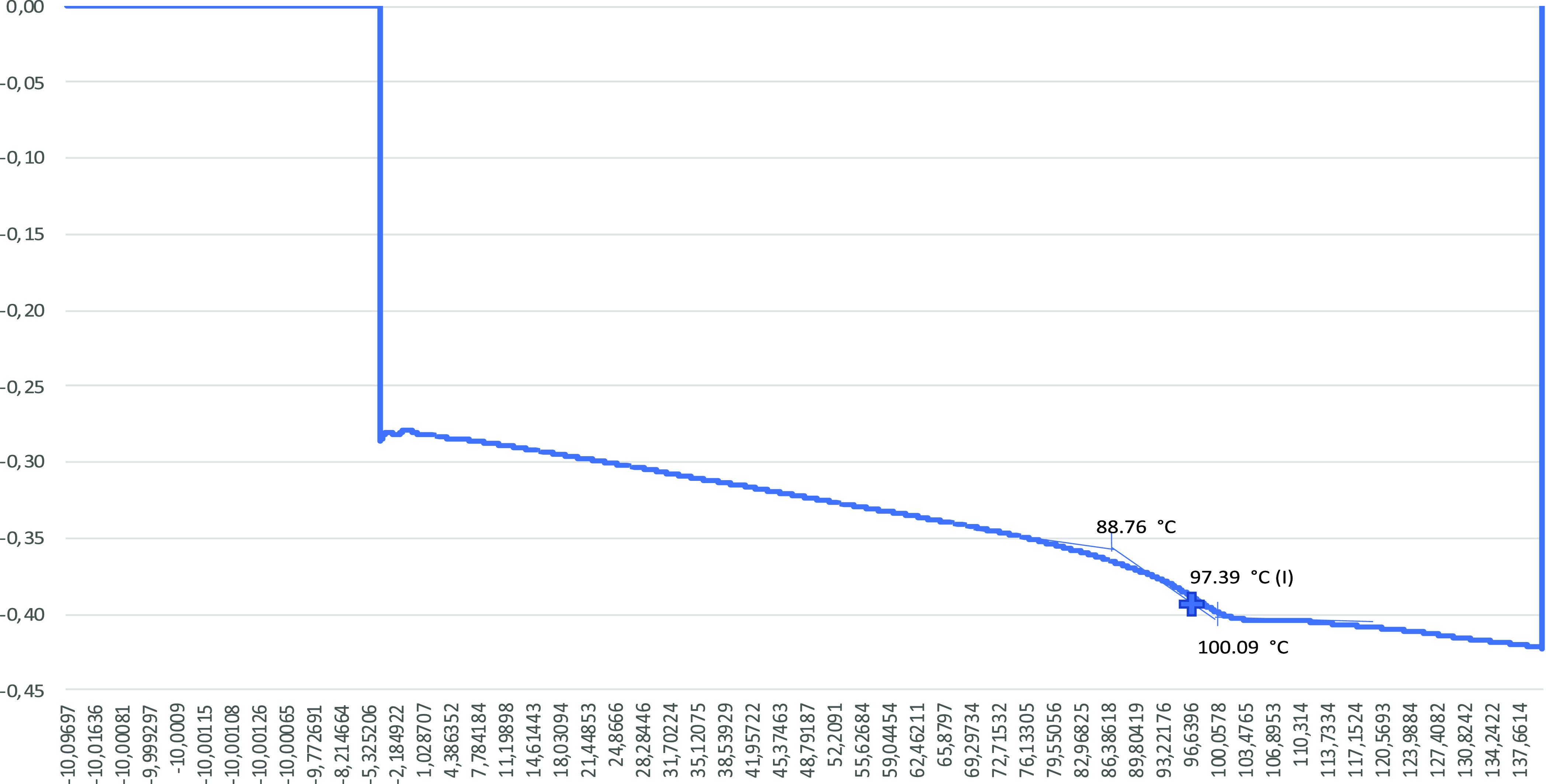
DSC graph for the 1% alkaline lignin included sample.

**Figure 6:**
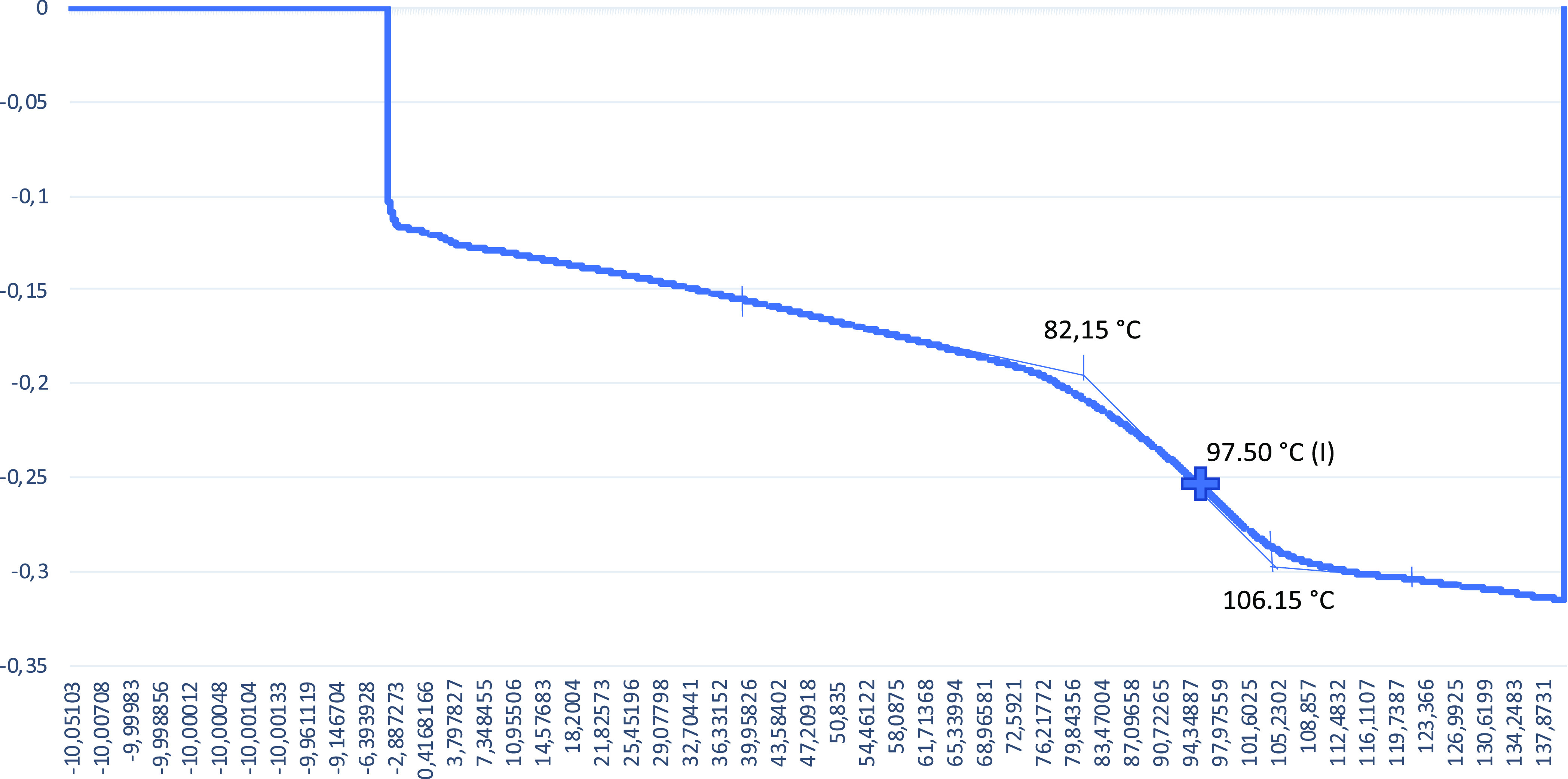
DSC graph for the 3% alkaline lignin included sample.

**Figure 7:**
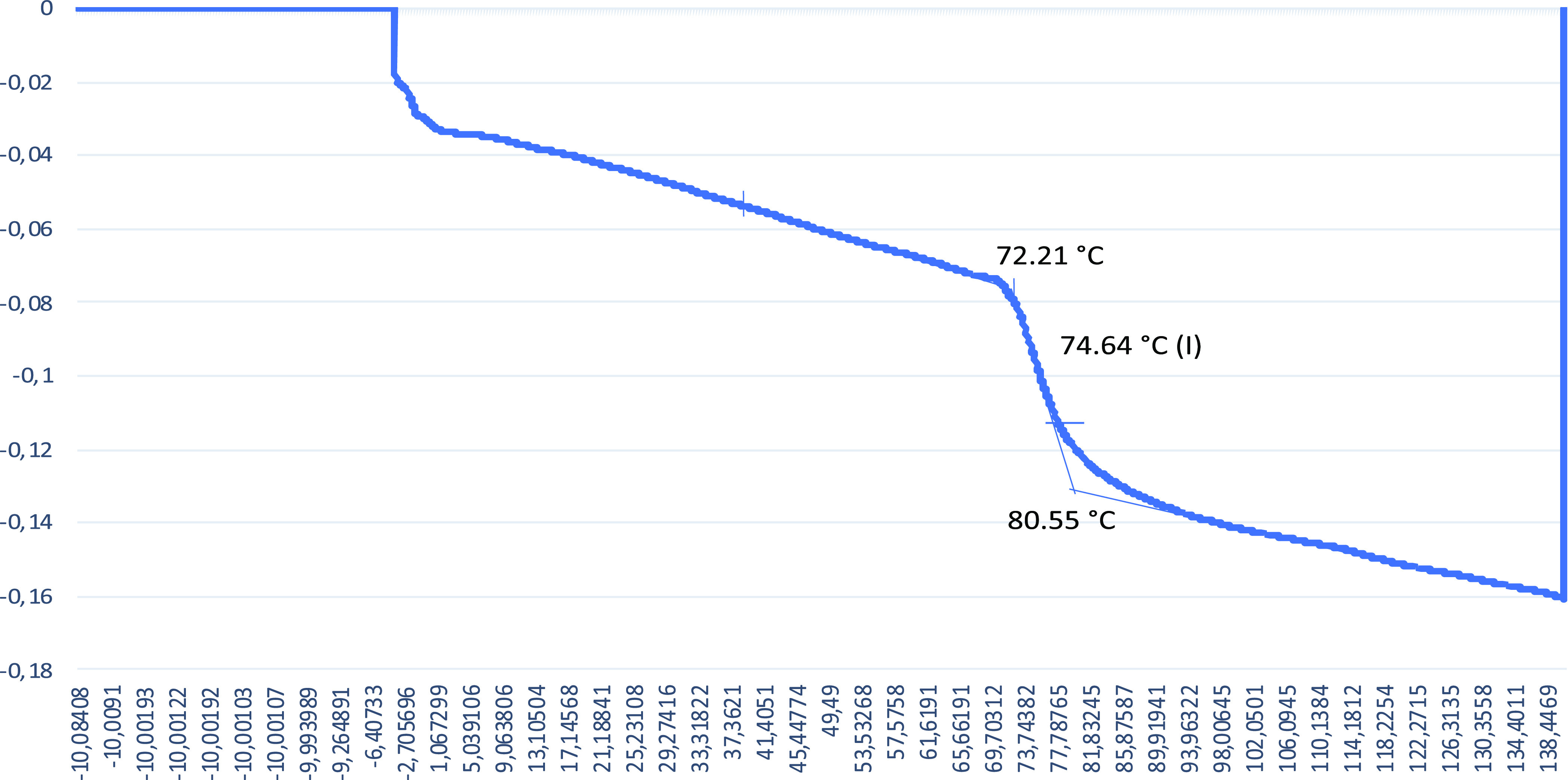
DSC graph for the 5% alkaline lignin included sample.

The *T*_g_ value for the 0% alkaline lignin included sample is 77.93 °C. With increasing alkaline lignin ratio, the *T*_g_ value is increased to 97.39 and 97.50 °C for 1 and 3% alkaline lignin ratios, respectively. Alkaline lignin added to the polymer structure at low rates of 1 and 3% resulted in an increase in the *T*_g_ temperature of the produced material thanks to the hard alkaline lignin segments in the shape memory polymer structure.^51^

However, increasing alkaline lignin to 5% resulted in a decrease in the *T*_g_ (74.64 °C) value of the SMPCs. This result can be attributed to the plasticizing effect of alkaline lignin addition of more than 3% and the reduction of intermolecular interaction by increasing the distance between the chains of the shape memory polymer to which it is added.^52^ Increasing the distance between the chains of the polymer raises molecular mobility. This is attributed to the amount of alkaline lignin making the polymer, consequently the SMPCs, more ductile due to chemical bondings.^53^

The increase in *T*_g_ temperature of the material with 1 and 3% alkaline lignin supplementation is thought to be due to the formation of secondary hydroxyl bonds, which act as semicrosslinks and somewhat restrict the movement of long chain molecules. With 5% alkaline lignin supplementation, alkaline lignin exerted a plasticizing effect and caused a decrease in *T*_g_ temperature.^45^

### 4.2 DMA Analysis

The viscoelastic properties of the samples are analyzed with the DMA device. In this regard, storage modulus, loss modulus, and tan delta values are obtained. Storage modulus indicates the behaviors of the samples in the elastic region. The loss modulus is a measure of the energy exerted by the material as heat, under load at high temperature. Tan delta is known as the damping factor, and the peak of tan delta is a point of transition to viscous region.

The storage modulus (*E*’) is known as a good parameter to determine the elastic component of viscoelastic materials. When storage modulus values are investigated, 0% alkaline lignin included SMPCs have 7300 MPa storage modulus. The storage modulus for 1 and 3% alkaline lignin included samples is 8900 and 8500 MPa, respectively. The 5% alkaline lignin included sample has 9000 MPa storage modulus. This confirms that the increased lignin content increases the ductile behavior of the material.^54^

The storage modulus results showed that the storage modulus of the sample containing 5% alkaline lignin was higher than the other ratios. With alkaline lignin supplementation above a certain ratio, the plasticizing effect of alkaline lignin becomes more pronounced. With this plasticizing effect, the plastic deformation ability of the material is increased, which causes an increase in the storage modulus of the material. This shows that alkaline lignin makes shape memory epoxy resins more ductile.^55,56^

*T*_g_ values are determined using loss modulus and tan delta peaks. When the obtained *T*_g_ values from loss modulus peaks are determined, the *T*_g_ value for the 0% alkaline lignin included sample is 98.02 °C. While there is an increase in *T*_g_ values for 1 and 3% alkaline lignin ratios, it is seen that they still have *T*_g_ values close to the sample containing 0% alkaline lignin. However, increasing the alkaline lignin ratio up to 5% showed a decrease of the *T*_g_ value to 88.19 °C. Increasing the alkaline lignin ratio up to 5% resulted in a decrease in crosslinking in the polymer structure. For this reason, the *T*_g_ value of the sample is decreased. Therefore, DMA results are found to be compatible with DSC results.

The loss modulus represents the energy loss caused by the internal friction caused by the movement of the polymer chains. The maximum loss modulus increased from 1200 to 1500 MPa as the lignin content increased from 0 to 5% by weight, indicating that the addition of alkaline lignin to the epoxy resin increases the mobility of the polymer chains during the glass transition. This increment in loss modulus can also be explained by decreasing crosslinking densities with increasing lignin content in the shape memory epoxy resin, similar to the decrease in *T*_g_ temperature.^55^

This suggests that the rigid and highly branched structure of lignin imparts both reinforcing and toughening effects to the obtained shape memory polymer composite; it is seen that the presence of a branched structure of alkaline lignin in the shape memory polymer, especially at the rate of 5% alkaline lignin, has a toughening effect on the polymer structure and causes a more ductile structure.

The effect of the addition of alkaline lignin on the ductile behavior in the shape memory polymer chain is seen as a result of the addition of alkaline lignin above 3%. As can be seen from the results of the DSC analysis, alkaline lignin supplementation above 3% increases the ductile behavior in the shape memory polymer chain and decreases the *T*_g_ temperature of the material. The decrease in the tan delta value seen in the DMA graph of SMPC containing 5% alkaline lignin also confirms that the addition of alkaline lignin above 3% reduces the *T*_g_ temperatures by the plasticizing effect on the composite material. In addition, the fact that the tan delta of the composite material containing 5% alkaline lignin is broader indicates that there is relaxation in the polymer structure.^52^ In this regard, the 5% alkaline lignin included sample has the best storage modulus and broader tan delta curve. Thus, it has competitive viscoelastic properties with the 0% alkaline lignin included sample.

This shows that the alkaline lignin included in the shape memory polymer in different ratios has an effect on the molecular bonds formed in the mixture, and the hydrogen bonds formed between the alkaline lignin and anhydride in the shape memory polymer mixture containing higher alkaline lignin cause a decrease in the strong crosslinks in the mixture. This was interpreted as the increase in plasticizing effect as a result of the addition of alkaline lignin (5%) to the shape memory polymer above a certain ratio. This creates low-strength bonds and increases the molecular distance as it reduces crosslinking bond formation.

The crosslinking densities were calculated and are presented in Table 1 to show the effect of alkaline lignin (0, 1, 3, 5%) included in the shape memory polymer mixture at different rates on the crosslinking formation in the shape memory polymer mixture.

**Table 1:** Crosslinking Densities of 0, 1, 3, and 5% Alkaline Lignin Included Samples

sample	*E*′ (MPa)	*T*_g_ (°C)	ρ (mol/cm^3^)
0%	404	78	0.046
1%	730	97	0.079
3%	759	97	0.082
5%	500	74	0.039

The crosslinking density (ρ) of a cured epoxy mixture is directly proportional to the storage modulus in the rubbery region and can be calculated by the formula below, according to the rubber elasticity theory.^55^
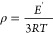
*R*, gas constant, 8.314 J/mol K; *E*′, the storage modulus in the rubbery region (at *T*_g_ + 30 °C); *T*, absolute temperature at *T*_g_.

As can be seen from Table 1, in alkaline lignin supplementation made at low rates such as 1 and 3%, the cross-linking densities increased as a result of the inclusion of hard lignin segments in the shape memory polymer mixture. In 5% alkaline lignin supplementation, the branched structure of lignin increases the distance between the shape memory polymer mixture molecules and causes a decrease in cross-linking formation. It is also seen from the results of cross-linking density that alkaline lignin, which has a hard and branched structure together, has a reinforcing and toughening effect on the sample.

**Figure 8:**
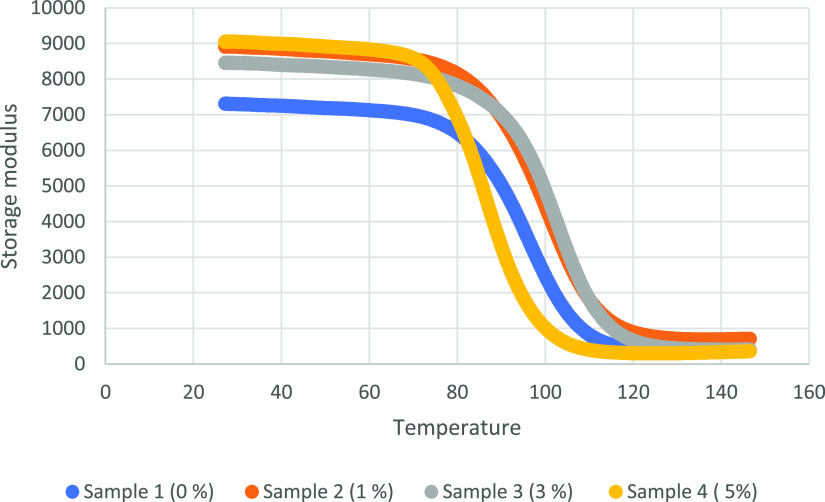
Storage modulus graphs of four samples.

Compared with pure shape memory epoxy resin, lignin-based epoxy resins showed improvement in storage modulus in the glassy region (storage modulus) with increasing lignin content, while an opposite trend was observed in the rubbery region (*T*_g_ + 30 °C). It was thought that 5% alkaline lignin supplementation, compared to other ratios, increased storage modulus in the glassy region, and the plasticizing effect of alkaline lignin caused by the distribution of the branched structure of alkaline lignin in the shape memory epoxy matrix. Even though alkaline lignin is modified, it is less reactive than commercial epoxy resin during the curing process. This causes less complete crosslinking and lowers the crosslinking density.^57^ DMA graphs for 0, 1, 3, and 5% alkaline lignin included SMPCs are shown in Figures 8^9^,10.

**Figure 9:**
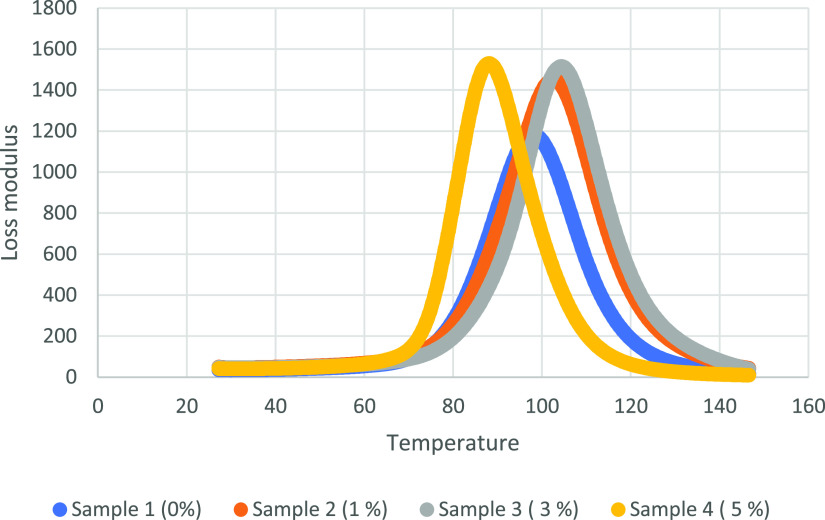
Loss modulus graphs of four samples.

**Figure 10:**
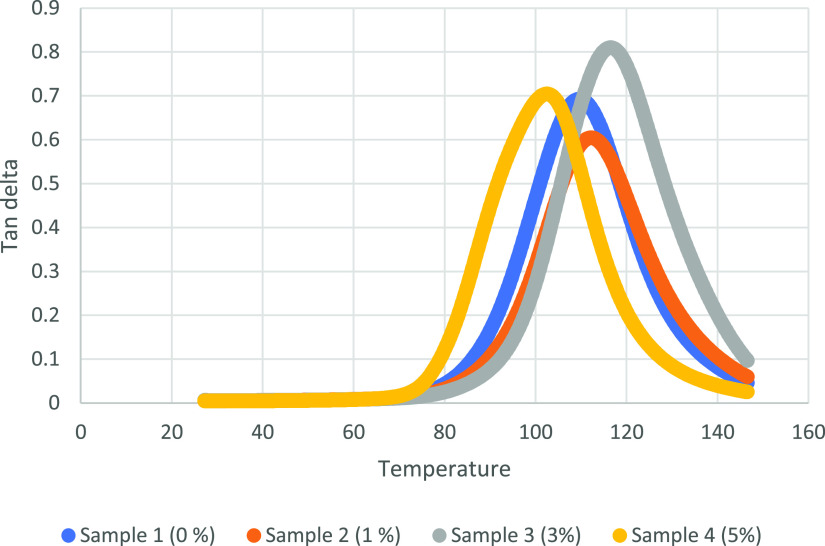
Tan delta graphs for four samples.

The plasticizing effect of alkaline lignin included in the shape memory polymer structure becomes more pronounced by 5%, and the effect of the weak intermolecular bonds formed by the shape memory polymer mixture is seen from the increase in the loss modules and expansion in the tan delta peaks. Loss modulus (*E*”) shows the energy dissipation as a result of the internal friction caused by the mobility of the polymer chains. It is seen that the loss modulus of the samples raises as the lignin ratio increases, indicating that the incorporation of alkaline lignin into the epoxy resin increases the ductile behavior in the shape memory polymer chains during the glass transition region.

### 4.3 SEM/EDS Analysis

SEM analysis is performed to determine the morphological properties and distribution of the alkaline lignin in the matrix phase.

**Figure 11:**
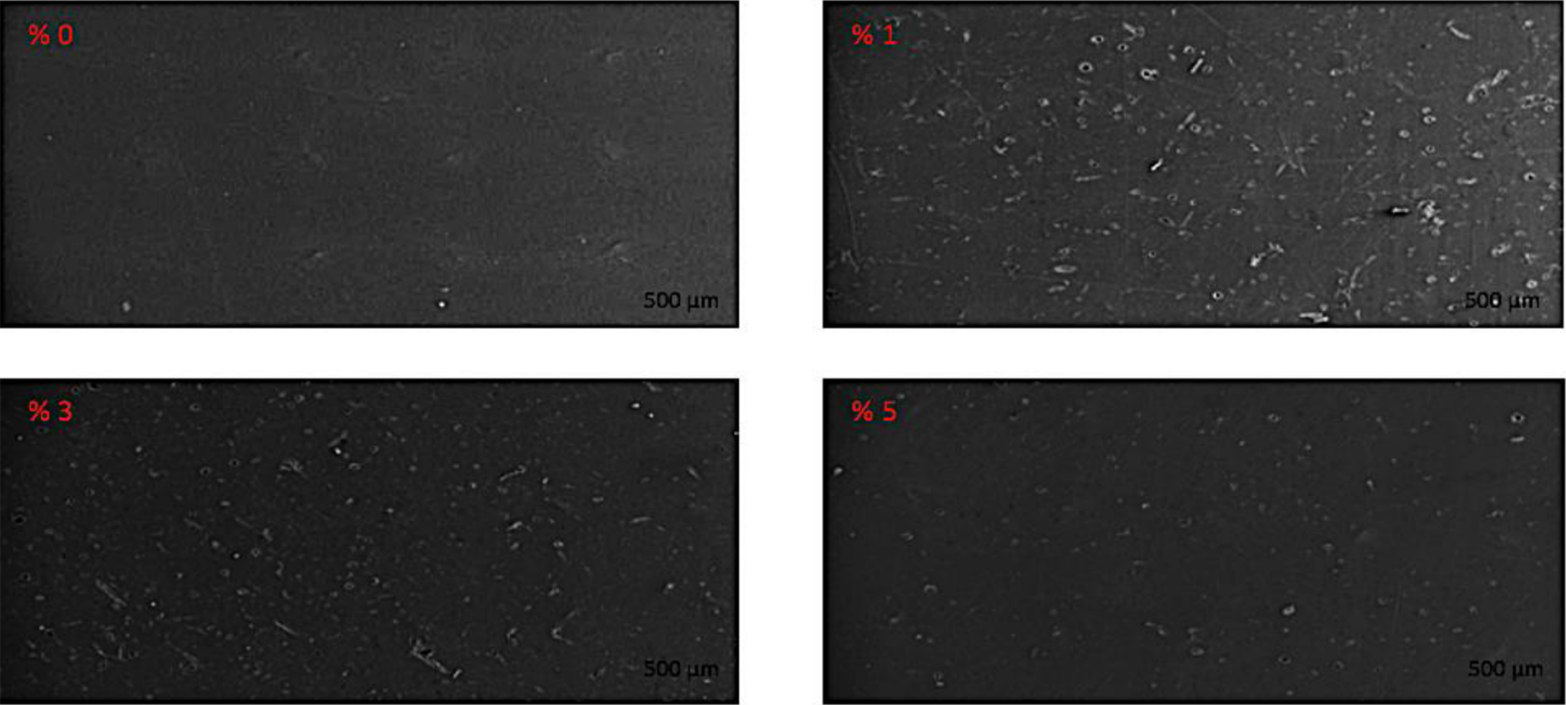
SEM images for four samples at 500 μm.

SEM results in Figure 11 show the incorporation of 1 and 3% alkaline lignin into the SMP structure, agglomerations, and fibril outs in the surface. However, 5% alkaline lignin is dispersed in the SMP structure, while less agglomeration is observed in 1 and 3% alkaline lignin included samples.

**Figure 12:**
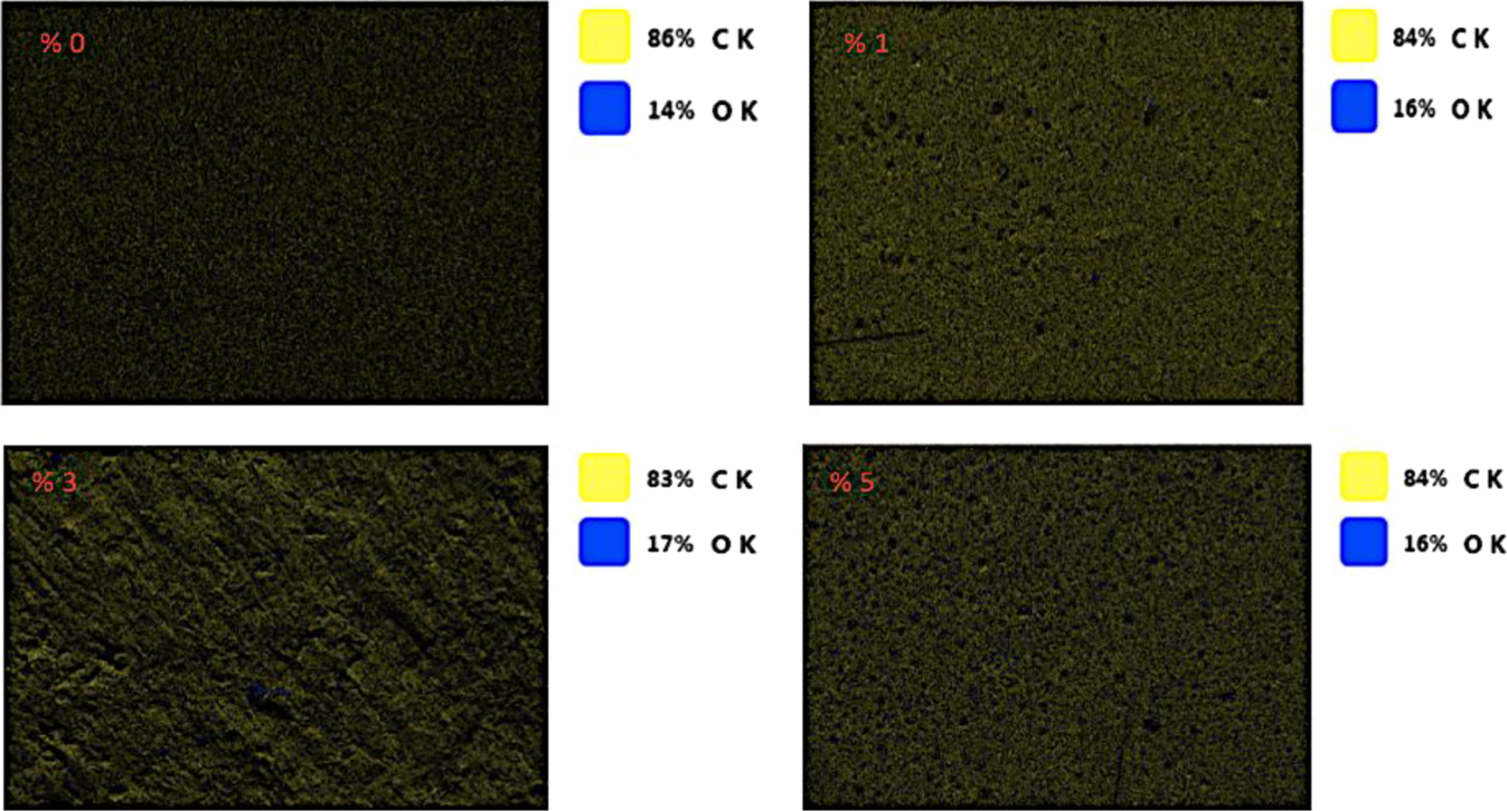
EDS images for four samples.

According to EDS analysis, it was observed that the samples containing 1, 3, and 5% alkaline lignin had carbon contents (83–84%) close to that of the sample without alkaline lignin (86%). EDS results in Figure 12 show that the epoxy resin mixture did not cause a significant decrease in the carbon content due to the carbon-based chemical composition of the alkaline lignin.

When the results obtained from the SEM analysis are examined, it is seen that the samples show roughness at 1 and 3% alkaline lignin content, while the samples tend to be smoother by increasing the lignin content to 5%, indicating the interaction between the shape memory polymer and alkaline lignin molecules.

While the fiber separations of the samples containing 1 and 3% alkaline lignin are more prominent, less fiber separation is seen in the sample with 5% alkaline lignin. A possible reason for greater fiber separation in composites containing 1 and 3% alkaline lignin may be phase separation due to lower lignin contents compared to 5% alkaline lignin-reinforced composites. In the 5% alkaline lignin-included composites, more molecular interaction occurred between the shape memory polymer mixture and the alkaline lignin, possibly due to the higher lignin content (5%).^44,45^

Since the −OH groups of alkaline lignin, which have hydrophilic properties, increase in the shape memory polymer mixture, it causes the formation of hydrogen bonds with the anhydride in the shape memory polymer mixture. These bonds, which are formed as a result of this molecular interaction between the shape memory polymer mixture and the alkaline lignin with 5% alkaline lignin supplementation, cause the formation of hydrogen bonds with lower strength than the crosslinking bonds.

According to the results obtained from the SEM analysis, better dispersion was obtained in the polymer matrix at the rate of 5% alkaline lignin, which caused the material to have a better flexural modulus. On the other hand, the presence of more alkaline lignin in the polymer structure increased the ductile behavior with the toughening effect that decreased the degree of cross-linking. Therefore, the composite material with a 5% alkaline lignin ratio had a lower *T*_g_ temperature.

Supplementation of alkaline lignin above a certain rate leads to an increase in the interaction between the aliphatic and aromatic groups of alkaline lignin and the shape memory polymer mixture. Alkaline lignin, which is known to be less reactive than the shape memory polymer and has a hydrophilic structure, causes the formation of low strength bondings (hydrogen and van der Waals interaction) with anhydride in the shape memory polymer mixture, which are known to be less strong than crosslinking bonds. This shows that alkaline lignin, which has a reinforcing and toughening effect on the sample, has a toughening effect on the sample above a certain ratio (5%).

With the toughening effect of alkaline lignin included in the structure, it causes an increase in the plastic deformation properties of the materials. These results consistent with the DMA and three-point bending test results indicate that increasing alkaline lignin content provides a more ductile structure.

Different levels of lignin agglomeration results in the formation of different microspheres. It is seen that lignin agglomerations occur in a spherical form depending on the ratios used. Irregularly shaped deformations and voids are also seen in the SEM images of samples with 1 and 3% alkaline lignin content. This indicates that there is a stronger polymer-filler interaction in the polymer matrix. These strong polymer-filler interactions resulted in an increment in the *T*_g_ values. The increase of the *T*_g_ values for 1 and 3% alkaline lignin can be explained by the alignment of the solubility properties and the possibility of polar–polar interaction. Compatibility of 1 and 3% alkaline lignin with DGEBA resulted in an increase in *T*_g_ of the obtained composites. The addition of 5% alkaline lignin resulted in a smoother surface morphology due to their plasticization effect.

In alkaline lignin-added SMP mixtures, brittle fractures and parabolic signs are observed. Interestingly, it appears that the size of the parabolic marking increases with increasing alkaline lignin ratio. The parabolic marking indicates that the alkaline lignin is effectively intertwined with the SMP matrix. These parabolic structures, which are more prominent in the SEM images of the sample containing 5% alkaline lignin and represent a more intertwined structure, also represent the ductile structure obtained as a result of 5% alkaline lignin supplementation. Furthermore, the good bending properties of the sample with 5% alkaline lignin content confirm that the alkaline lignin is effectively intertwined with the SMP matrix and the material has a more ductile structure.

The pure epoxy resin shows a relatively smooth fracture surface without any ductility, except for some lines typical of brittle material. In addition to this, samples containing 1 and 3% alkaline lignin showed a rougher surface compared to the sample containing 0% alkaline lignin, indicating greater plastic deformation during fracture. Plastic deformation represents the energy absorption during rupture and it is thought that alkaline lignin, which has a plasticizing effect in the polymer structure, makes the epoxy resins more ductile and causes plastic deformation during fracture. While the samples containing 1 and 3% alkaline lignin showed a rough surface with unevenly dispersed particles, a less porous structure was obtained in the sample containing 5% alkaline lignin, as the molecular interaction between alkaline lignin and shape memory epoxy resin mixture increased. In addition, the toughening effect of alkaline lignin supplementation of 5% can also be seen in the image of the 5% alkaline lignin included sample.^58^

The SEM results of the samples are consistent with the results from the three-point bending tests. In other words, the inclusion of alkaline lignin into the shape memory polymer mixture at a high ratio (5%) improved the ductility of the resulting shape memory epoxy resin mixture. This resulted in an increase in storage modulus and bending modulus of the obtained alkaline lignin reinforced SMPCs.^55^

### 4.4 FTIR Analysis

FTIR analyses were carried out in order to observe the bonds formed between the alkaline lignin and shape memory polymer mixture molecules. From the FTIR results obtained, it was seen that the visible peaks of the samples containing 1, 3 and 5% alkaline lignin were almost the same, but their densities were different. This showed that the changing alkaline lignin ratio has an effect on the bonds between the shape memory polymer and the alkaline lignin mixture.

FTIR spectra are shown in Figures S1–S4. The presence of OH stretching vibrations in aromatic and aliphatic OH groups is observed in a wide absorption band at 3300 cm^–1^. While no peak is observed in the region representing the stretching vibrations of the −OH bonds of the sample containing 0% alkaline lignin, the −OH band is seen at 3300 cm^–1^ in samples containing 1, 3 and 5% alkaline lignin. From the FTIR spectrum, characteristic peaks assigned to alkaline lignin are seen in all samples with 1, 3, and 5% alkaline lignin ratios. The strong and broad peak centered at 3300 cm^–1^, ranging from 3000–3650 cm^–1^, represents the stretching vibration of O–H, which is mainly present in the form of hydrogen bonds between the molecules of the alkaline lignin and epoxy resin mixture. From here, it is seen that the peaks representing the −OH bond increase with the increasing lignin ratio.^54,59^

In the FTIR analysis, the strong signal seen at 1730 cm^–1^ representing the C–O stretch in the ester groups (aromatic and aliphatic) indicates esterification between the hydroxyl groups in the alkaline lignin structure and the epoxy resin mixture.^47,60^ As can be seen from the FTIR spectrum, it is seen that the ester bonds expected to form between the shape memory polymer mixture and alkaline lignin are formed.

The intensity of the C=O peak at 1730 cm^–1^, representing the molecular bonds between alkaline lignin and epoxy resin, increased for 1 and 3% alkaline lignin content.^54^ However, for the 5% alkaline lignin ratio, it was observed that the peak density representing the esterification reaction decreased at 1730 cm^–1^.

In the FTIR spectra, it is seen that the samples containing 1, 3, 5% alkaline lignin show −OH peaks, but the peak densities are different. It is seen that the peak density of −OH in the sample containing 1 and 3% alkaline lignin is lower than the sample containing 5% alkaline lignin. This was due to increased hydrogen bonding between alkaline lignin and anhydride in the shape memory polymer mixture as a result of increased alkaline lignin supplementation.

### 4.5 Three-Point Bending Tests

The stress and strain values are obtained from the three-point bending test results. The results show that bending modulus rises with increased alkaline lignin ratios. In DMA results, the storage modulus values are increased with the raising alkaline lignin ratio. This confirms that alkaline lignin supplementation causes an increase in storage modulus, which is a measure of the flexibility of the material. Similarly, the results obtained from three-point bending tests show that increasing alkaline lignin ratio causes an increase in the bending modulus of the material as shown in Table 2. Therefore, adding alkaline lignin into the structure enable SMPCs to have a more ductile structure.

**Table 2:** Three-Point Bending Test Results

sample	thickness (mm)	width (mm)	maximum force (N)	flexural modulus (MPa)
0%	2.35	11.75	348.3	388.89
1%	2.76	11.74	629.93	516.0
3%	2.91	11.76	672.41	497.63
5%	2.83	11.81	684.61	530.60

Adding alkaline lignin into the SMPs results in an improvement in flexural modulus as it can fill most of the cracks produced at the interface between the polymer matrix and the reinforcement element.^36,40^ Thanks to the hydrogen bonds in the structure of lignin, it forms hydrogen bonds with the polymer and increases the mechanical strength of the material. These results show that the alkaline lignin supplement, which is made at a low ratio (1 and 3%), provides good mixing with the polymer and creates a strong interface. However, it is seen that alkaline lignin supplementation above 3% increases the ductile behavior of the polymer and decreases the *T*_g_ temperature due to its plasticizing effect. Although this situation reduces the *T*_g_ of the material, the flexural modulus and mobility of the material increase.

Tanase-Opedal et al. reported an improvement in the ductility of the composite material by adding up to 10% lignin to the polymer.^35^ In the present study, the increased lignin content also made the material ductile; therefore, the sample with 5% alkaline lignin content had the highest bending modulus.

The porous structure seen at 1 and 3% is due to the hardening effect of the alkaline lignin addition. Since plastic deformation caused by alkaline lignin supplementation is a significant energy-absorbing process, it provides an increase in the amount of energy required for the formation of new surfaces in the material morphology. The increased molecular interaction between alkaline lignin/polymer as a result of 5% alkaline lignin supplementation results in an increase the material toughness. Although hydrogen bonds are formed between the polymer/alkaline lignin increased alkaline lignin supplementation, it is known that it reduces the cross-linking density in the polymer matrix. Therefore, 5% alkaline lignin supplementation decreases the *T*_g_ temperature of the material, while increasing the ductility of the material and increasing the flexural modulus.

At the ratio of 5% alkaline lignin, new bonds are formed as hydrogen bondings between the fiber/matrix and the material behaves more ductile in the inelastic region. Therefore, the elastic modulus value of the material increases. This ultimately results in higher load carrying capacity, especially in the region of plastic deformation.^61^

It can be concluded that alkaline lignin is covalently incorporated into the SMP matrix and it also adds heterogeneity to the homogeneous SMP matrix. However, the combination of tougher structure and lower crosslinking density due to lignin’s intrinsic heterogeneous nature exhibited a simultaneous reinforcing and toughening effect of the resulting epoxy resin mixtures.^62^ It is seen that the reinforcement of alkaline lignin increases the storage and flexural modulus of the material by increasing its toughness.

It is known that the excellent mobility of their molecular chains is one of the prerequisites for higher elongation at break of SMPCs. For this reason, 5% alkaline lignin supplement increases the elongation value at break by increasing the ductile behavior in the shape memory polymer chain. The sample with 5% alkaline lignin content shows plastic deformation due to the plasticizing effect of alkaline lignin supplementation.^63^ Plastic deformation represents the situation in which the stress hardly increases, even though the elongation value constantly goes beyond the yield point. It is seen that the 5% alkaline lignin ratio increases the interaction between alkaline lignin/SMP and 5% alkaline lignin supplementation increases molecular mobility and plastic deformation.

### 4.6 Shape Recovery Test Results

**Figure 13:**
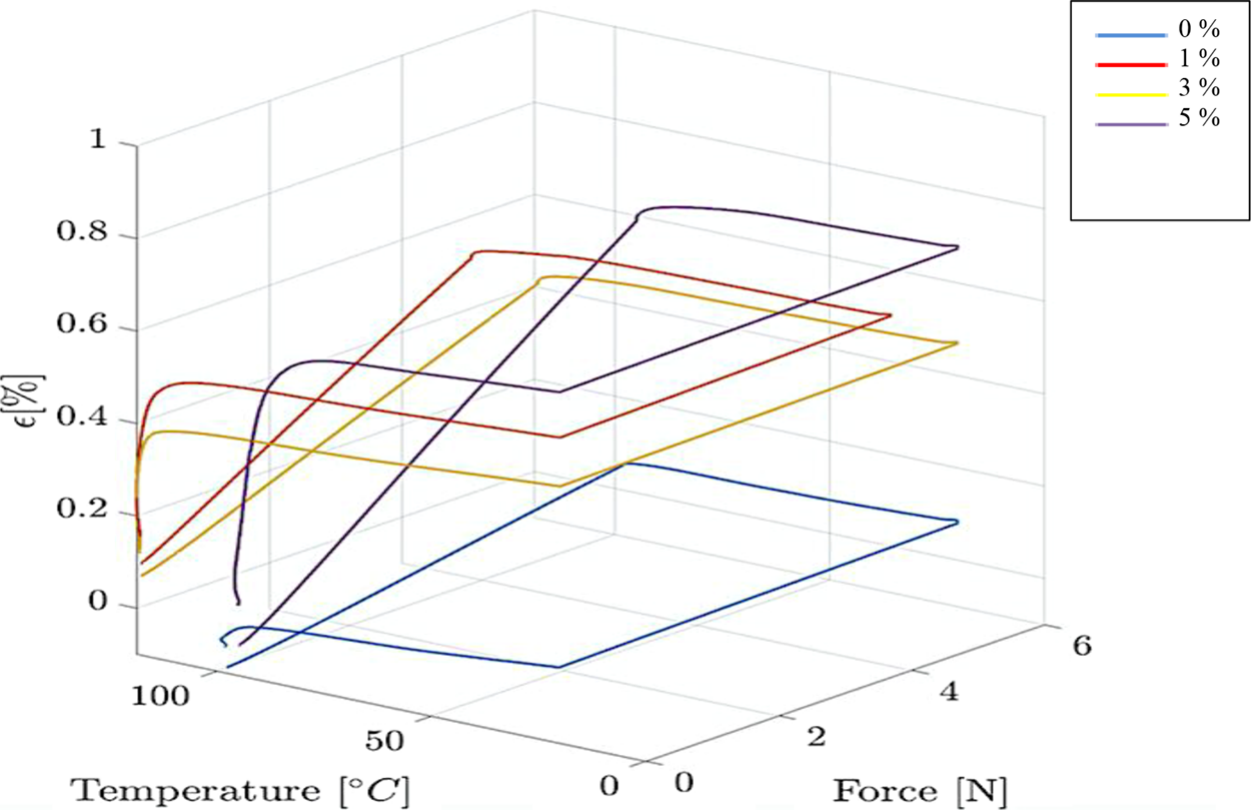
Strain–temperature–force diagram for four samples.

Strain-temperature-stress graphs of four samples are given in Figure 13. Shape fixity and recovery ratios are calculated by eqs 1 and 2 from shape recovery test results performed under the controlled force mode of the DMA (Q800) device.

The shape stability and shape recovery ratios of the samples are calculated by using the ε_max_, ε_fix_, and ε_r_ data obtained as a result of the analysis performed in the DMA device. The sample containing 0% alkaline lignin had 90% shape stability, while the samples containing 1, 3, and 5% alkaline lignin had 96–97% shape stability.

Additionally, when the shape recovery ratios are examined, the shape recovery ratio of the samples containing 0% alkaline lignin is 74%, while the ratio of 1 and 3% alkaline lignin samples was 76–77%, with a small increase. The shape recovery ratio of the sample containing 5% alkaline lignin was found to be 90%.

In 5% alkaline lignin supplementation, an increase in molecular chain mobility occurred due to the plasticizing effect of alkaline lignin. The thermo-mechanical test results show that the increased molecular chain mobility provides an increase in the bending modulus and storage properties of the sample. As a result, the increased lignin content improved the elastic behavior of the sample, resulting in an increase in the shape recovery ratio.

## 5 Conclusions

In this study, SMPCs were produced using alkaline lignin containing SMP and glass fiber, and thermo-mechanical, morphological, and shape memory tests of the produced SMPCs were performed. According to the results obtained from the DSC analysis, 3 and 5% alkaline lignin supplementation increases the *T*_g_ temperature of SMPC, while 5% alkaline lignin supplementation decreases the *T*_g_ temperature of SMPC. DMA analysis showed that the sample with the highest storage modulus is SMPC containing 5% alkaline lignin. While the increasing alkaline lignin ratio increased the loss modulus of SMPCs from 1200 to 1500 MPa, it was observed that the sample with the widest tan delta was SMPC with a ratio of 5%. As a result of the analyses, it was seen that the ratio of alkaline lignin supplementation on the bonds formed between molecules with alkaline lignin supplementation was effective and that these bonds were effective on the material structure. In 5% alkaline lignin supplementation, the branched structure of alkaline lignin becomes more prominent in the shape memory epoxy resin, causing intermolecular microdeformations and new low strength hydrogen bondings. These hydrogen bonds formed between alkaline lignin and anhydride in the shape memory polymer mixture, which is known to be less reactive than the shape memory polymer, were also confirmed from the FTIR spectrum. Furthermore, SEM analysis images also overlap with these results and show the rough and plastic deformation structure obtained by the distribution of alkaline lignin in the matrix phase. All of the results show that the rigid and highly branched structure of alkaline lignin imparts both reinforcing and toughening effects to the obtained shape memory polymer composite. The toughening effect of alkaline lignin is more prominent at 5% alkaline lignin supplementation, which causes an increase in the plastic deformation properties of the materials, resulting in a more ductile structure. As a result, it can be said that the toughening effect of alkaline lignin is more pronounced with the addition of 5% alkaline lignin, and this causes the plastic deformation properties of the materials to increase, resulting in a more ductile structure and thus the highest shape recovery ratio (90%). Overall, it can be concluded that the results were compatible with each other, and the alkaline lignin supplementation to the shape memory epoxy resin improved the stretching behavior, which is highly effective on the SMPC shape memory properties.

## Supporting Information Available

FTIR analysis results of SMPCs (PDF)
