# Supplementary material for: New Approach to Shape Memory Polymer Composite Production Using Alkaline Lignin-Reinforced Epoxy-Based Shape Memory Polymers
Source: ACS Omega. 2023 Apr 20;8(17):15003–16. doi: 10.1021/acsomega.2c07812 (PMC10157668; doi:10.1021/acsomega.2c07812)
Supplement: Supplementary file 1 — ao2c07812_si_001.pdf [file ao2c07812_si_001.pdf]

Supporting Information

A new approach to shape memory polymer composites production using alkaline lignin reinforced epoxy-based shape memory polymers

Merve Uyan[a,b], Melih Soner Celiktas[b*]

[a]Department of Mechanical Engineering, University of Alberta, Edmonton, Alberta, Canada

[b]Solar Energy Institute, Ege University, 35100 Bornova-Izmir, Turkey

*soner.celiktas@ege.edu.tr, merveuyn@gmail.com

FTIR analysis results of SMPCs: **Figure S1-S4** shows the FTIR spectrums of 0,1,3 and 5 % alkaline lignin included samples. The presence of -OH stretching vibrations in aromatic and aliphatic OH groups is observed in a wide absorption band at 3300 cm^{-1}. While no peak is observed in the region representing the stretching vibrations of the –OH bonds of the sample containing 0% alkaline lignin as shown in **Figure S1**, -OH band is seen at 3300 cm^{-1} in samples containing 1,3 and 5% alkaline lignin. From the FTIR spectrum, characteristic peaks assigned to alkaline lignin are seen in all samples with 1, 3 and 5% alkaline lignin ratios.

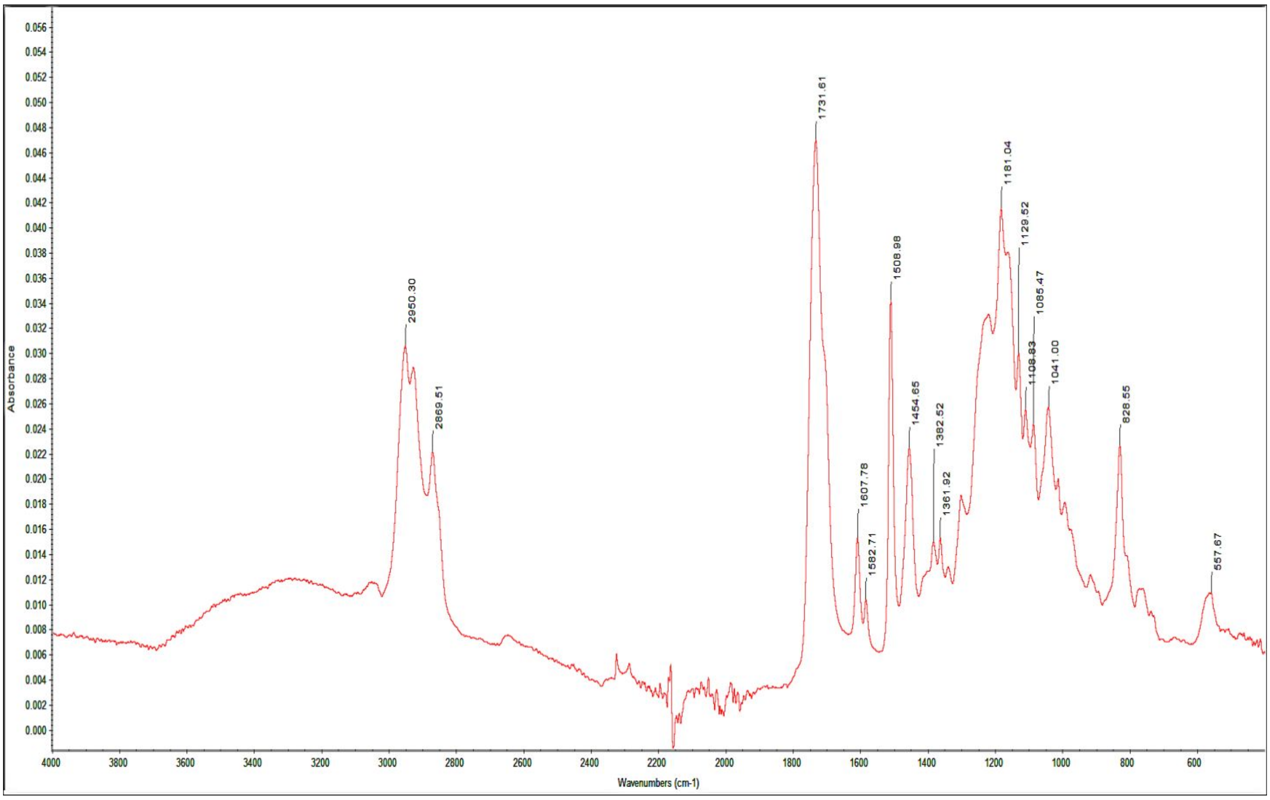

Figure S1: The FTIR spectrum for 0 % alkaline lignin included SMPCs.

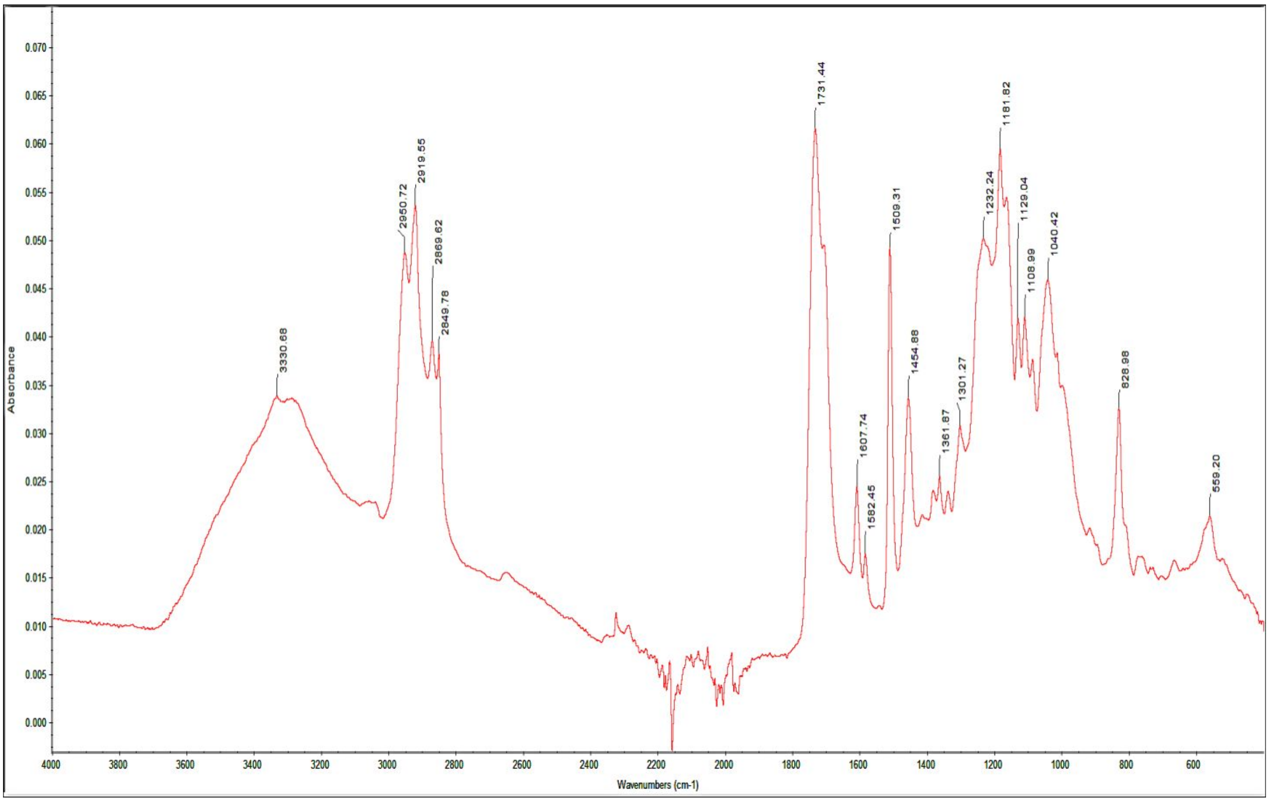

Figure S2: The FTIR spectrum for 1 % alkaline lignin included SMPCs.

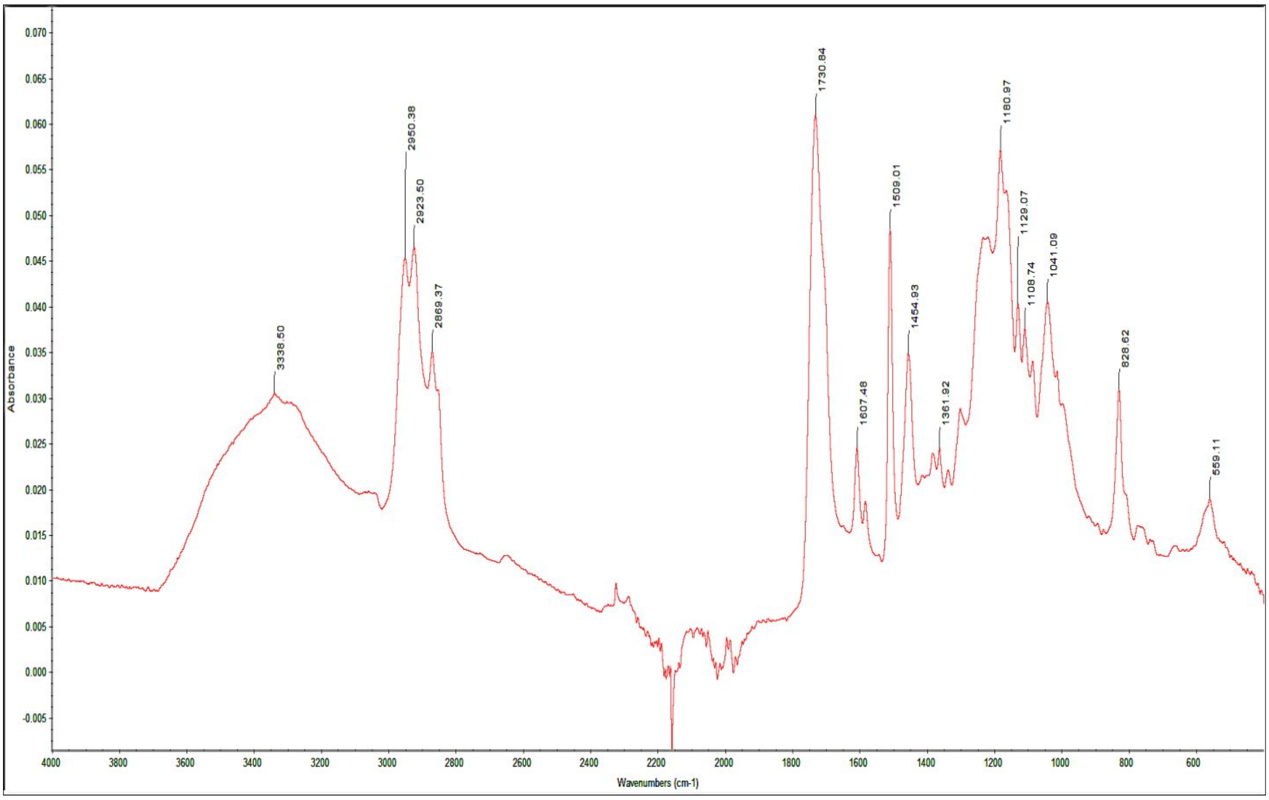

Figure S3: The FTIR spectrum for 3 % alkaline lignin included SMPCs.

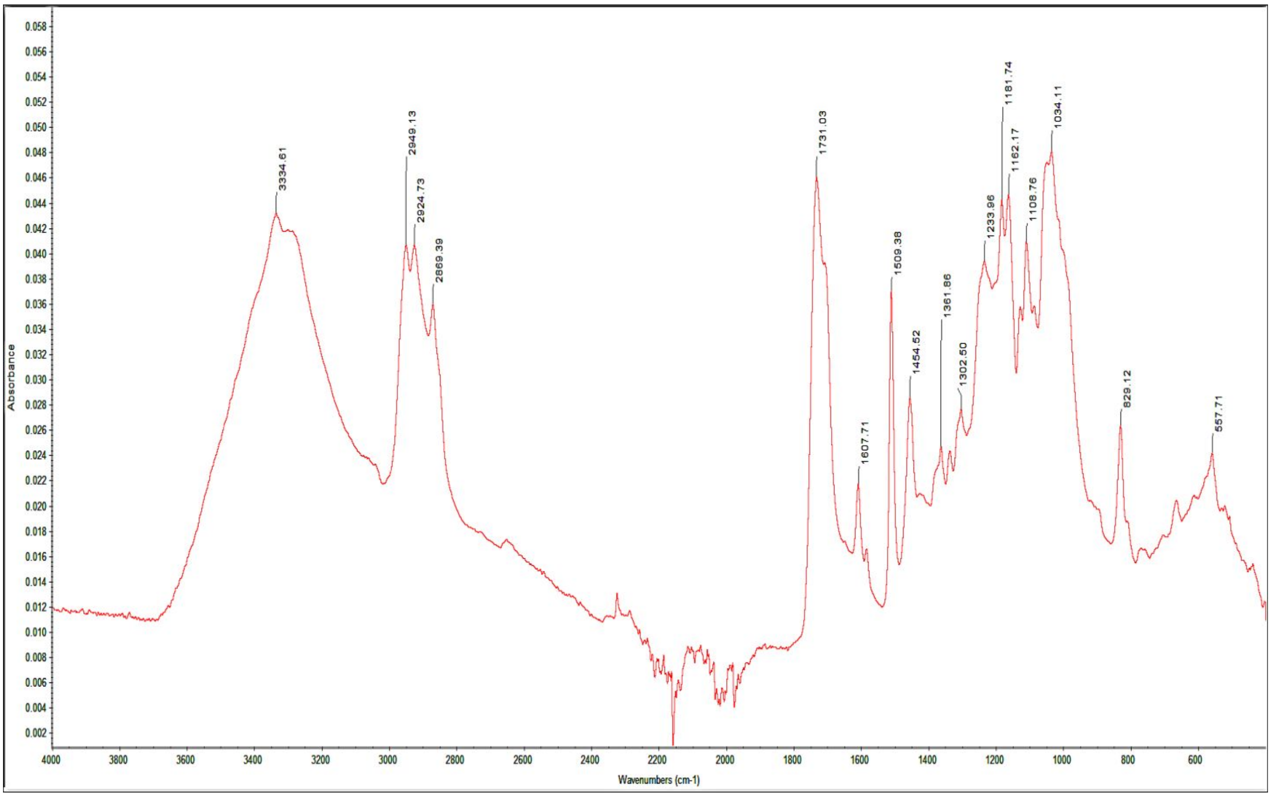

Figure S4: The FTIR spectrum for 5 % alkaline lignin included SMPCs.

Figure S2 and S3 shows that the intensity of the C=O peak at 1730 cm^{-1}, representing the molecular bonds between alkaline lignin and epoxy resin, increased for 1 and 3 % alkaline lignin content (Wang et al., 2018). However, for the 5% alkaline lignin ratio, it was observed in **Figure S4** that the peak density representing the esterification reaction decreased at 1730 cm^{-1}.
